# Congenital granular cell epulis in a neonate: a case report and review of diagnosis, treatment, and prognosis

**DOI:** 10.3389/froh.2025.1548291

**Published:** 2025-08-11

**Authors:** P. C. Guidone, Roberta Seccia, L. A. Fabrocini, G. Troiano, G. Maffei, Maria Carmela Pedicillo, Giuseppe Pannone, Lorenzo Lo Muzio, Rosanna Zamparese, Giorgio Mori, Ilenia Sara De Stefano

**Affiliations:** ^1^ Department of Oral Surgery, Policlinico Riuniti Foggia, Foggia, Italy; ^2^ Pathological Anatomy Unit, Department of Clinical and Experimental Medicine, University of Foggia, Foggia, Italy; ^3^ Department of Clinical and Experimental Medicine, University of Foggia, Foggia, Italy; ^4^ Department of Medicine and Surgery, LUM University, Casamassima, Italy; ^5^ Neonatology end Neonatal Intensive Care Unit, Policlinico Riuniti Foggia, Foggia, Italy; ^6^ Legal Medicine Unit, Ascoli Piceno Hospital C-G. Mazzoni, Ascoli Piceno, Italy; ^7^ Pathology Unit, Department of Clinical and Experimental Medicine, University of Foggia, Foggia, Italy

**Keywords:** benign tumors, congenital epulis, granular cell tumor, infancy tumor, oral tumor, histopathology, immunohistochemistry

## Abstract

Congenital granular cell epulis (CGCE) is a rare benign tumor typically presenting at birth and most commonly located on the maxillary alveolar ridge.

**Case Presentation:**

We report the case of a five-day-old female with a smooth, multilobulated mass on the right maxillary alveolar ridge causing feeding difficulties. Surgical excision was performed under sedation. Histological and immunohistochemical analysis confirmed the diagnosis of CGCE. No recurrence was observed at one-month follow-up.

**Conclusion:**

Early diagnosis and surgical treatment of CGCE are essential to avoid functional impairment. The prognosis is excellent following complete excision.

## 1 Introduction

Congenital granular cell epulis (CGCE) is a rare benign lesion that typically presents at birth or during early infancy, showing a marked female predominance (1, 2). It most frequently arises from the maxillary alveolar ridge and is characterized by the presence of large granular cells. Despite overlapping features with other oral granular cell lesions—such as adult granular cell tumor (GCT)—CGCE exhibits distinct histopathological and immunohistochemical properties, which are essential for its diagnosis (3, 4). Surgical excision is usually curative, and the prognosis is generally excellent (5).

## 2 Case report

### 2.1 Patient information

The patient, a five-day-old female neonate born at term after an uneventful pregnancy and delivery, with no associated congenital anomalies or relevant family history, was admitted to the Neonatal Intensive Care Unit due to the presence of a voluminous oral mass observed at birth.

### 2.2 Clinical findings

**Figure 1:**
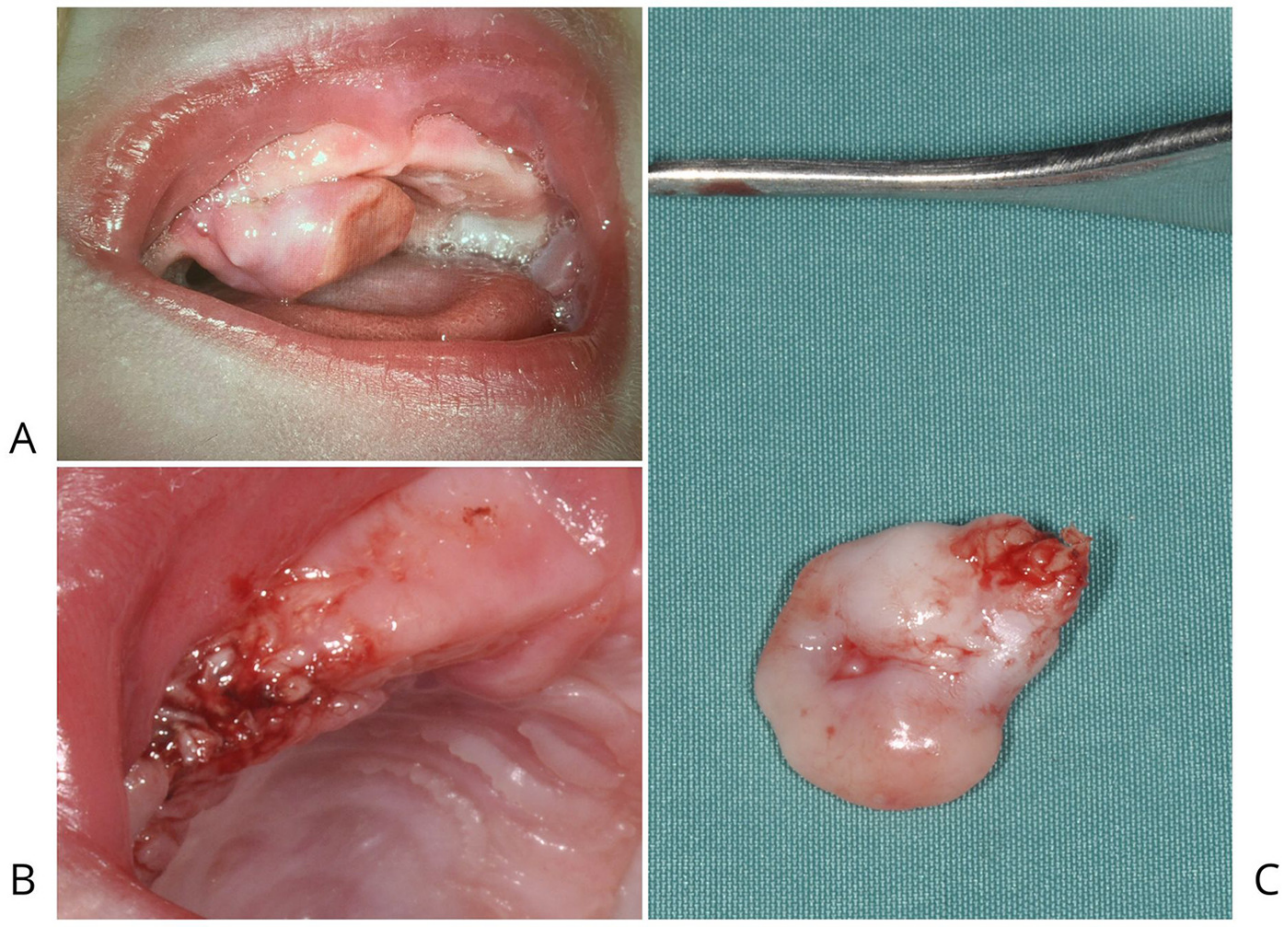
**(A)** CGCE attached to the right maxillary edentulous alveolar ridge by a pedicle, was responsible for causing significant feeding difficulties in the newborn. **(B)** Post-surgical image of the patient following electrosurgical excision of CGCE located on the alveolar portion of the right maxilla. **(C)** Smooth, pink, multilobulated mass located on alveolar portion of the right maxilla.

On clinical examination, the lesion appeared as a well-defined, smooth-surfaced, pink, and multilobulated mass arising from the right maxillary edentulous alveolar ridge, measuring approximately 1 cm in its greatest dimension. It was firm in consistency and attached by a pedunculated base, allowing partial mobility on palpation. The overlying mucosa was intact, with no evidence of ulceration, bleeding, or local inflammation (Figures 1A,B).

The mass was readily visible upon intraoral inspection and significantly interfered with normal feeding. During breastfeeding attempts, the lesion mechanically obstructed the infant's ability to latch and suck, causing ineffective feeding and nutritional concern. Despite the lesion's size and protrusion into the oral cavity, the neonate exhibited no signs of respiratory distress at rest, and oxygen saturation remained within normal limits. However, the potential for dynamic airway compromise during feeding or crying could not be excluded.

The lesion's location, clinical features, and age of onset were suggestive of congenital granular cell epulis (CGCE). However, other differential diagnoses considered at this stage included congenital hemangioma, lymphangioma, congenital teratoma, neuroectodermal tumor of infancy, and other rare oral soft tissue tumors of the neonate.

### 2.3 Diagnostic assessment

#### 2.3.1 Macroscopic examination

The excised lesion measured approximately 1.0 × 0.8 × 0.2 cm and appeared as a smooth, pink, and multilobulated mass (Figure 1C). Gross features were consistent with the clinical presentation of a benign soft tissue tumor arising from the alveolar ridge. The mass was well-circumscribed and exhibited elastic consistency without signs of ulceration or hemorrhage, supporting a preliminary diagnosis of congenital epulis with granular cell morphology.

#### 2.3.2 Histological examination

**Figure 2:**
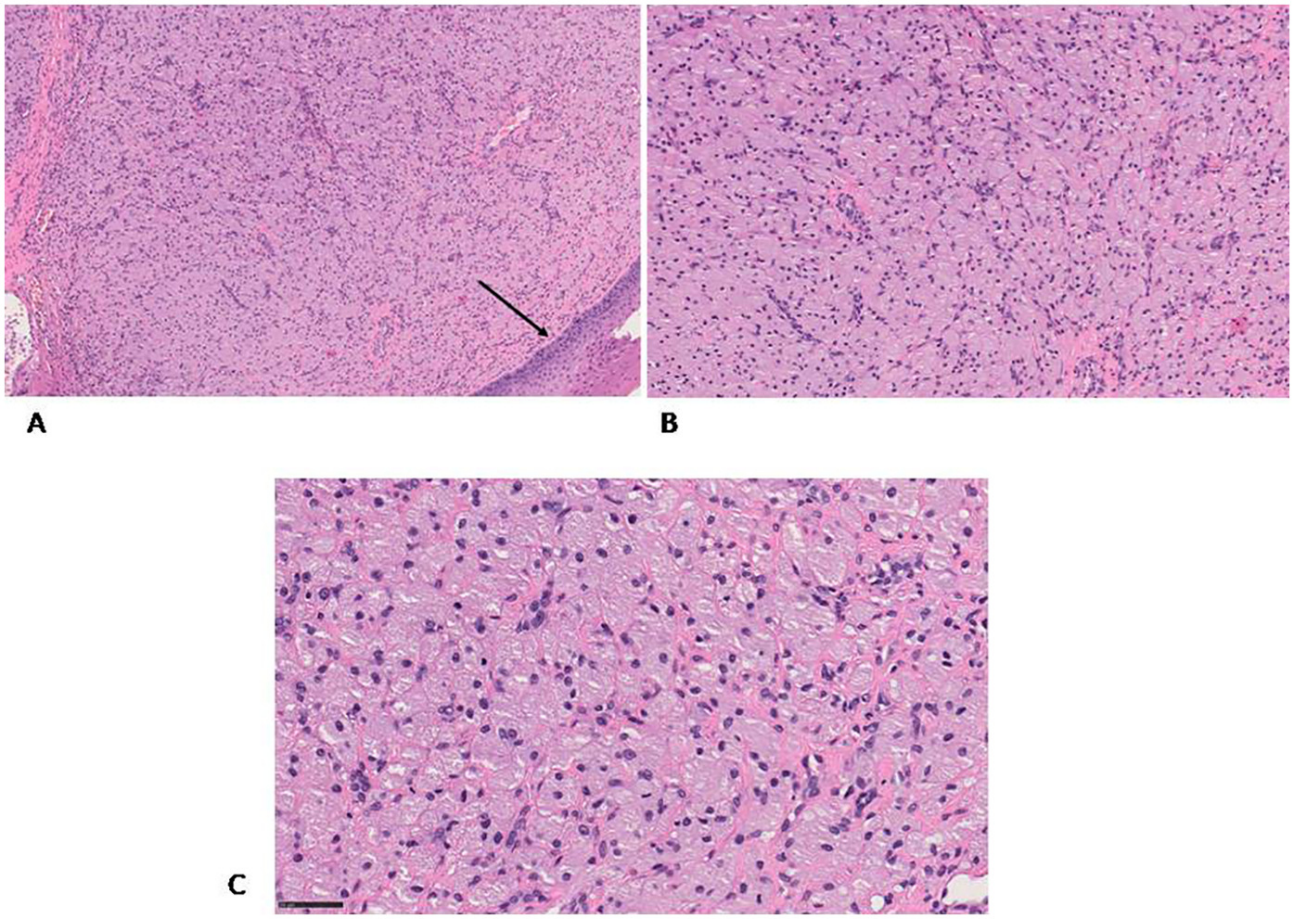
H&E staining **(A)** the granular cells are covered by flattened stratified squamous epithelium (black arrow) **(B,C)** the granular cells are well defined and contain an eccentric nucleus, they are arranged in a network of capillaries. Original magnification 10× **(A)**, 20× **(B)** and 40× **(C)** NanoZoomer S60 C13210 series.

**Figure 3:**
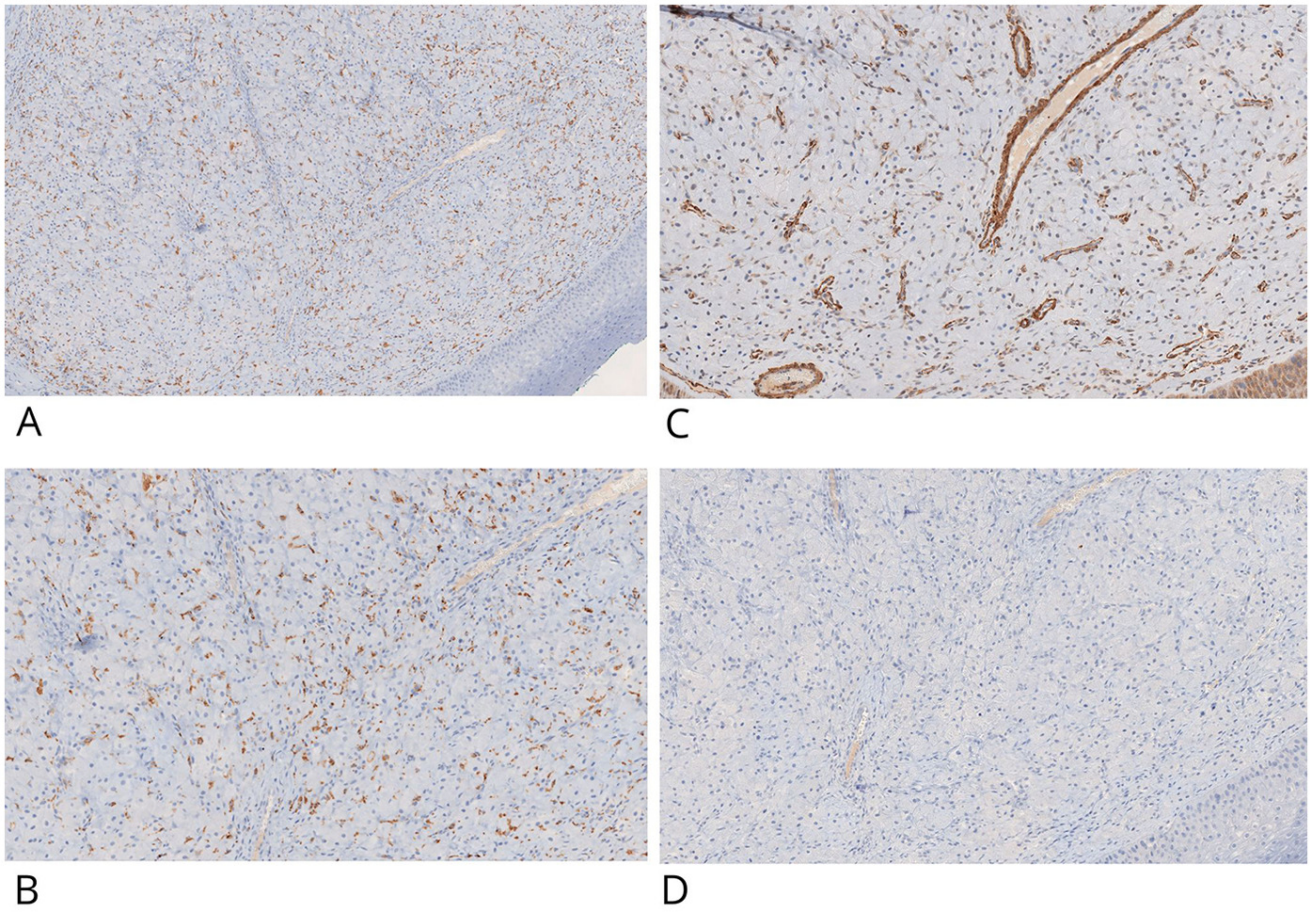
IHC positive staining of CD68 in inflammatory histiocytic elements and negative staining in granular cells (CD68-) **(A,B)**. Granular cells showed no IHC staining potential for actin-smooth muscle **(C)** and desmin **(D)** Original magnification 10× **(A)** and 20× **(B–D)** ×20 NanoZoomer S60 C13210 series.

The specimen was fixed in 10% neutral buffered formalin, embedded in paraffin, and sectioned at 4 μm thickness. Sections were placed on non-coated glass slides and stained with hematoxylin and eosin (H&E) for standard histopathological evaluation (Figure 2). Microscopy revealed a proliferation of large polygonal cells with abundant granular eosinophilic cytoplasm and centrally located small nuclei. These cells were arranged in sheets within a well-vascularized stroma containing a dense capillary network. Scattered histiocytic inflammatory elements, positive for CD68, were observed in the background (Figures 3A,B).

#### 2.3.3 Immunohistochemical analysis

**Figure 4:**
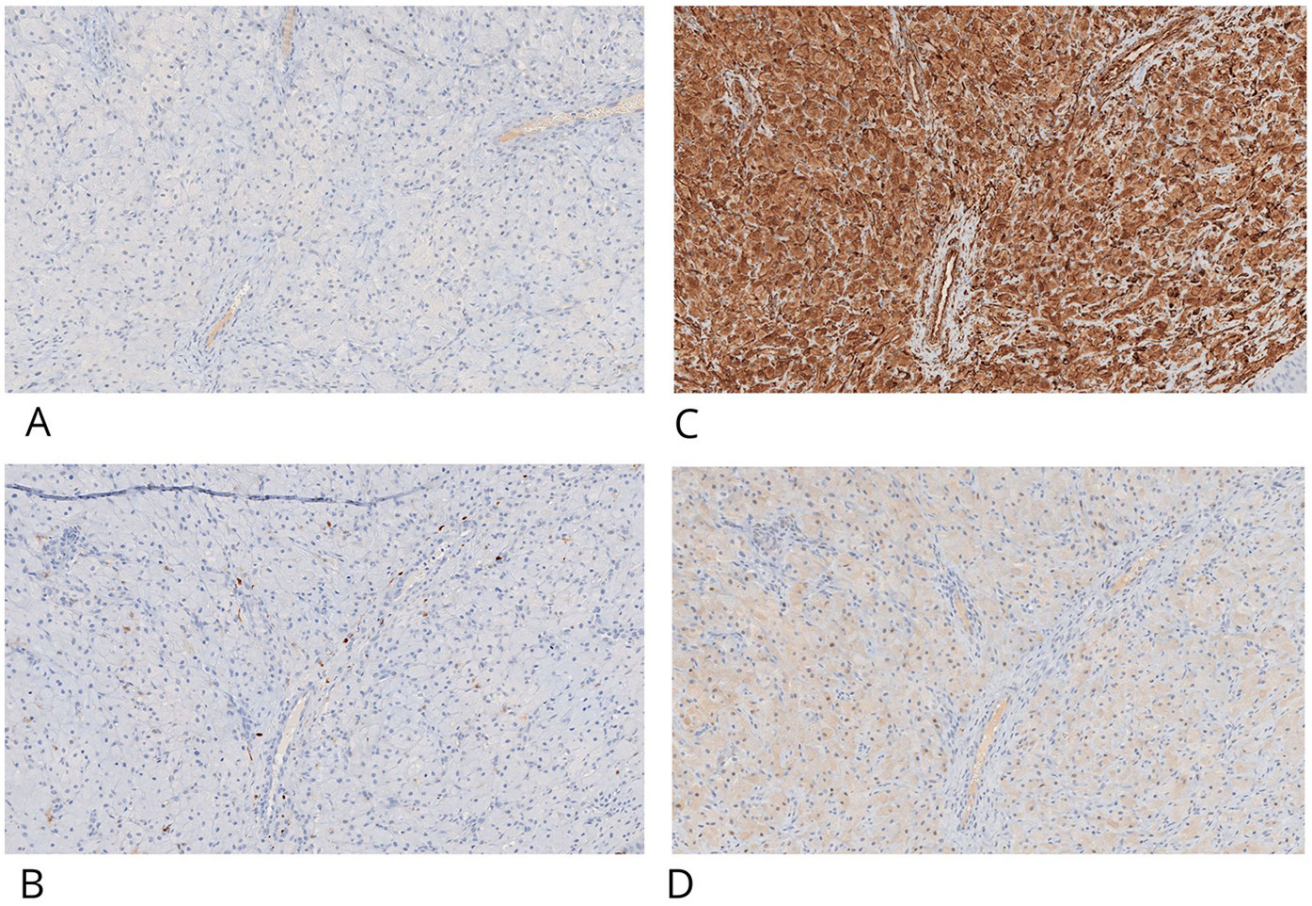
CGCE showed negative IHC staining for CD-1a **(A)** and S100, light positive is present in interstitial cells **(B)** granular cells are positive for IHC staining for vimentin **(C)** and neuron-specific enolase- NSE **(D)** original magnification ×20 nanoZoomer S60 C13210 series.

Immunohistochemistry (IHC) was performed on 4 μm paraffin-embedded sections mounted on poly-L-lysine-coated glass slides, using the linker streptavidin-biotin horseradish peroxidase (LSAB-HRP) method on the Ventana Benchmark XT autostainer. The following primary antibodies (Ventana/Roche, prediluted) were employed: smooth muscle actin (clone 1A4), desmin (clone DE-R11), vimentin (clone V9), CD68 (clone KP-1), CD1a (clone EP3622) (Figures 3C,D, 4A,B), neuron-specific enolase (NSE, clone MRQ-55) (Figures 4C,D), and S100 (polyclonal). Negative control slides were included to ensure antibody specificity.

Immunohistochemical evaluation, performed by an expert surgical pathologist using an Olympus BX41 optical microscope and NanoZoomer S60 C13210 digital scanner, confirmed the diagnosis. The granular cells showed strong cytoplasmic positivity for vimentin and NSE, and were negative for S100, cytokeratin AE1/AE3, smooth muscle actin, desmin, and CD1a. CD68 positivity was limited to scattered background histiocytes and not present in the granular cell component. This combined histological and immunohistochemical profile is diagnostic for congenital granular cell epulis, clearly distinguishing it from granular cell tumors of neural origin.

#### 2.3.4 Therapeutic intervention

A prompt surgical dental consultation was undertaken. Based on the multidisciplinary assessment, the decision was made to proceed with early surgical excision under sedation using electrosurgical technique, both to relieve functional obstruction and to obtain tissue for definitive histopathological diagnosis.

#### 2.3.5 Follow-up and outcomes

Residual electrocoagulative effects were observed at the surgical resection margin, characterized by localized tissue changes indicative of thermal injury. The patient underwent a follow-up examination one month after the surgical procedure. Clinical assessment demonstrated excellent healing of the surgical site, with no signs of infection, dehiscence, or other postoperative complications. The mucosal and tissue regeneration appeared optimal, indicating effective wound healing and satisfactory tissue integration.

#### 2.3.6 Patient perspective

The patient's parents expressed satisfaction with the clinical outcome and appreciated the prompt diagnosis and treatment provided.

#### 2.3.7 Informed consent

Written informed consent was obtained from the patient's legal guardians for publication of this case report and accompanying images.

## 3 Discussion

Histologically, CGCE is composed of large polygonal cells with granular eosinophilic cytoplasm and small, bland nuclei, typically arranged in nests or sheets within a vascular stroma (6). These features resemble adult granular cell tumors (GCTs), which also affect the oral cavity—most commonly the tongue—and are more frequently observed in women aged 30–60 years (7) (Figure 5 Supplementary Data).

However, important differences exist between CGCE and GCT. The most significant differential diagnostic factor lies in their **immunohistochemical profile**. GCTs are typically **S100-positive**, reflecting their Schwannian origin, while CGCE generally lacks S100 expression (Figure 6 Supplementary Data) (7). Furthermore, **CD68 expression**, typically strong and diffuse in GCTs due to their lysosome-rich cytoplasm, is often weak or absent in CGCE (Figure 7 Supplementary Data) (8, 9). In the present case, immunohistochemical analysis confirmed these expected patterns, with CGCE cells testing negative for S100, cytokeratin AE1/AE3, desmin, smooth muscle actin, and CD1a, but positive for **vimentin** and **neuron-specific enolase (NSE)**.

Another key histological distinction lies in the relationship with the overlying epithelium: **GCTs frequently show pseudoepitheliomatous hyperplasia (PEH)**, a reactive epithelial proliferation that can mimic squamous cell carcinoma, whereas CGCE typically lacks this feature (10, 11). The absence of PEH in CGCE further supports its benign and distinct nature.

From a **clinical standpoint**, although CGCE is benign, its presence can interfere with essential functions such as breastfeeding, as observed in our case. In rare instances, spontaneous regression has been documented (12), but in most cases, **conservative surgical excision** is the preferred management approach due to potential mechanical obstruction or aesthetic concerns. Recurrence after complete excision is exceedingly rare (13).

While the **etiopathogenesis** of CGCE remains unclear, occasional familial cases suggest a possible genetic predisposition (14). Further research is needed to elucidate the **molecular pathways** involved in its development and to better define its origin within the spectrum of congenital oral lesions (15).

Finally, the **postoperative outcome** in this case showed excellent healing, without complications. This is in line with recent studies indicating that optimal wound healing depends on factors such as the surgical technique, tissue characteristics, and postoperative care (16, 17).

## Data Availability

The original contributions presented in the study are included in the article/Supplementary Material, further inquiries can be directed to the corresponding author.
