# Supplementary material for: Congenital granular cell epulis in a neonate: a case report and review of diagnosis, treatment, and prognosis
Source: Front Oral Health. 2025 Aug 11;6:1548291. doi: 10.3389/froh.2025.1548291 (PMC12375573; doi:10.3389/froh.2025.1548291)
Supplement: Supplementary file 1 [file Datasheet1.pdf]

Supplementary files

Fig.5: H&E. Granular cell tumors found in adult (GCT)Original magnification 10x (A), 20x (B) and 40x (C)NanoZoomer S60 C13210 series

Fig. 6: Fig.3: IHC positive staining of S100. GCT is typically S-100 positive, indicating neural origin. Original magnification 20x NanoZoomer S60 C13210 series

Fig. 7: IHC positive staining of CD68. GCTs consistently show strong and diffuse CD68 positivity due to their abundant lysosome-rich cytoplasm, supporting a Schwannian origin. Original magnification 20x NanoZoomer S60 C13210 series
