# Supplementary figures and images for: Congenital granular cell epulis in a neonate: a case report and review of diagnosis, treatment, and prognosis
Source: Front Oral Health. 2025 Aug 11;6:1548291. doi: 10.3389/froh.2025.1548291 (PMC12375573; doi:10.3389/froh.2025.1548291)

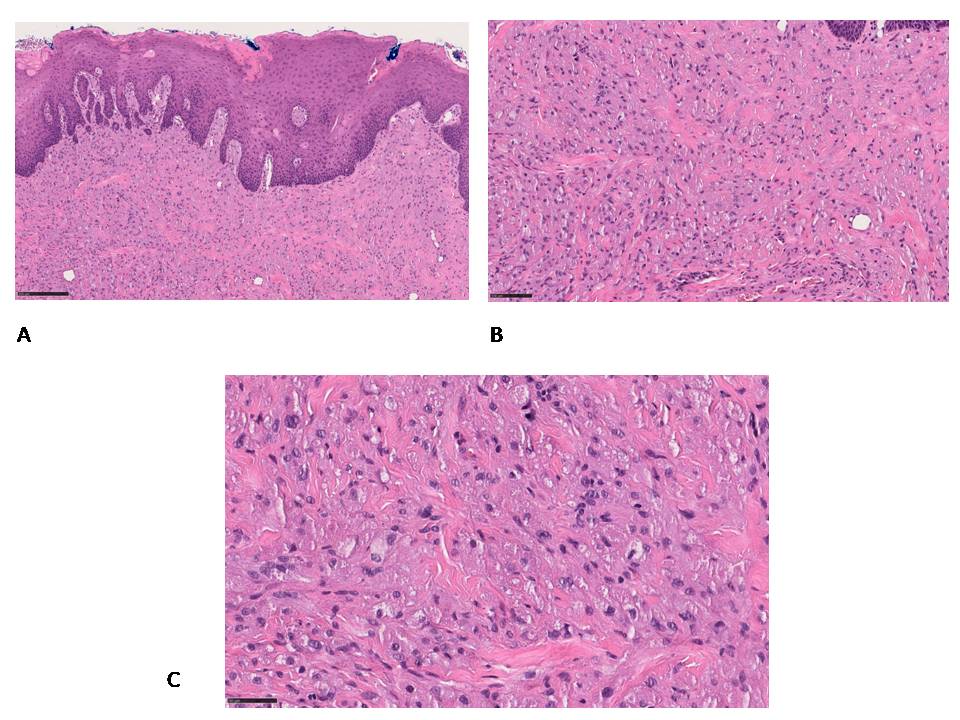

Supplement: Supplementary file 3 [file Image1.jpeg]

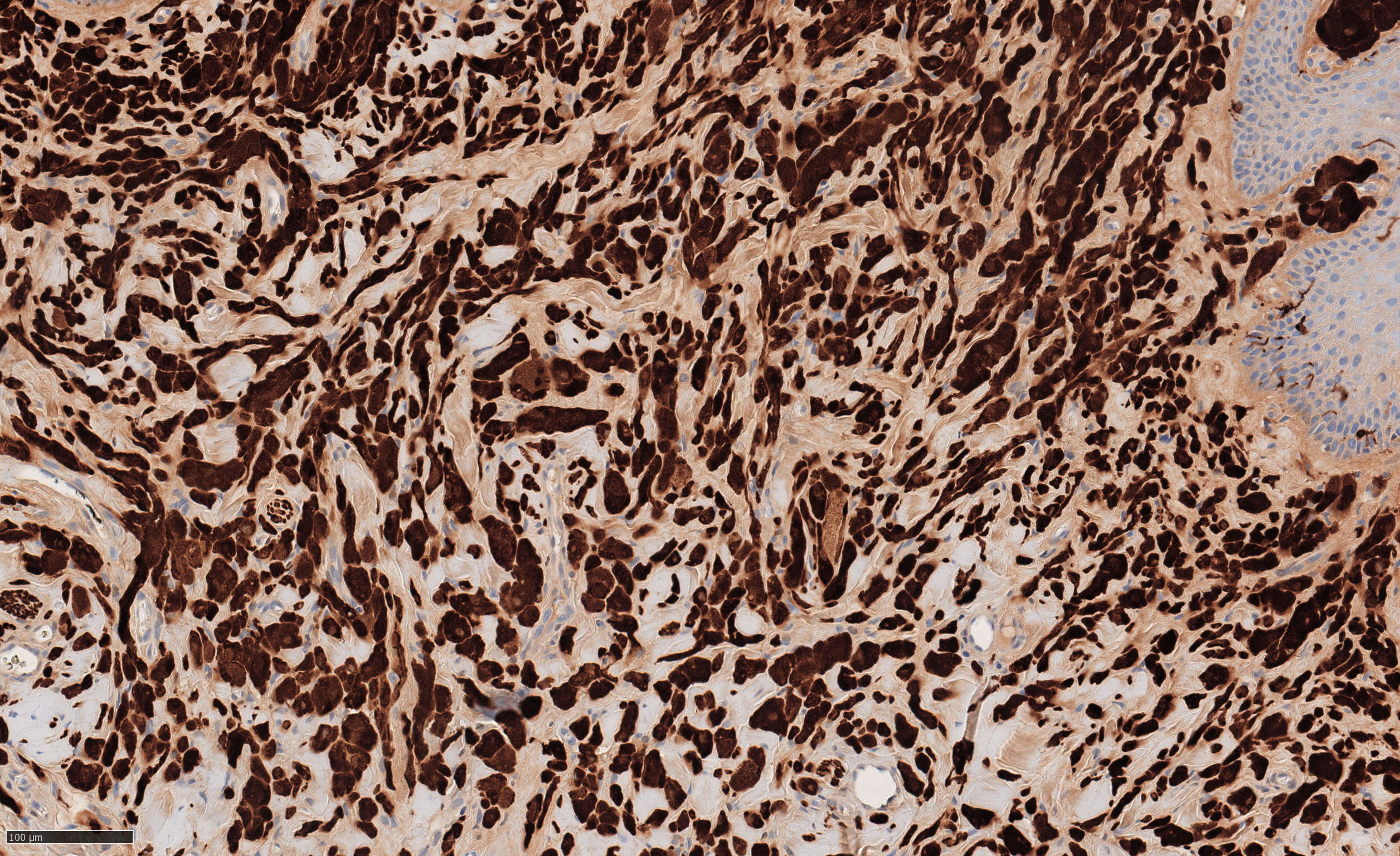

Supplement: Supplementary file 4 [file Image2.jpeg]

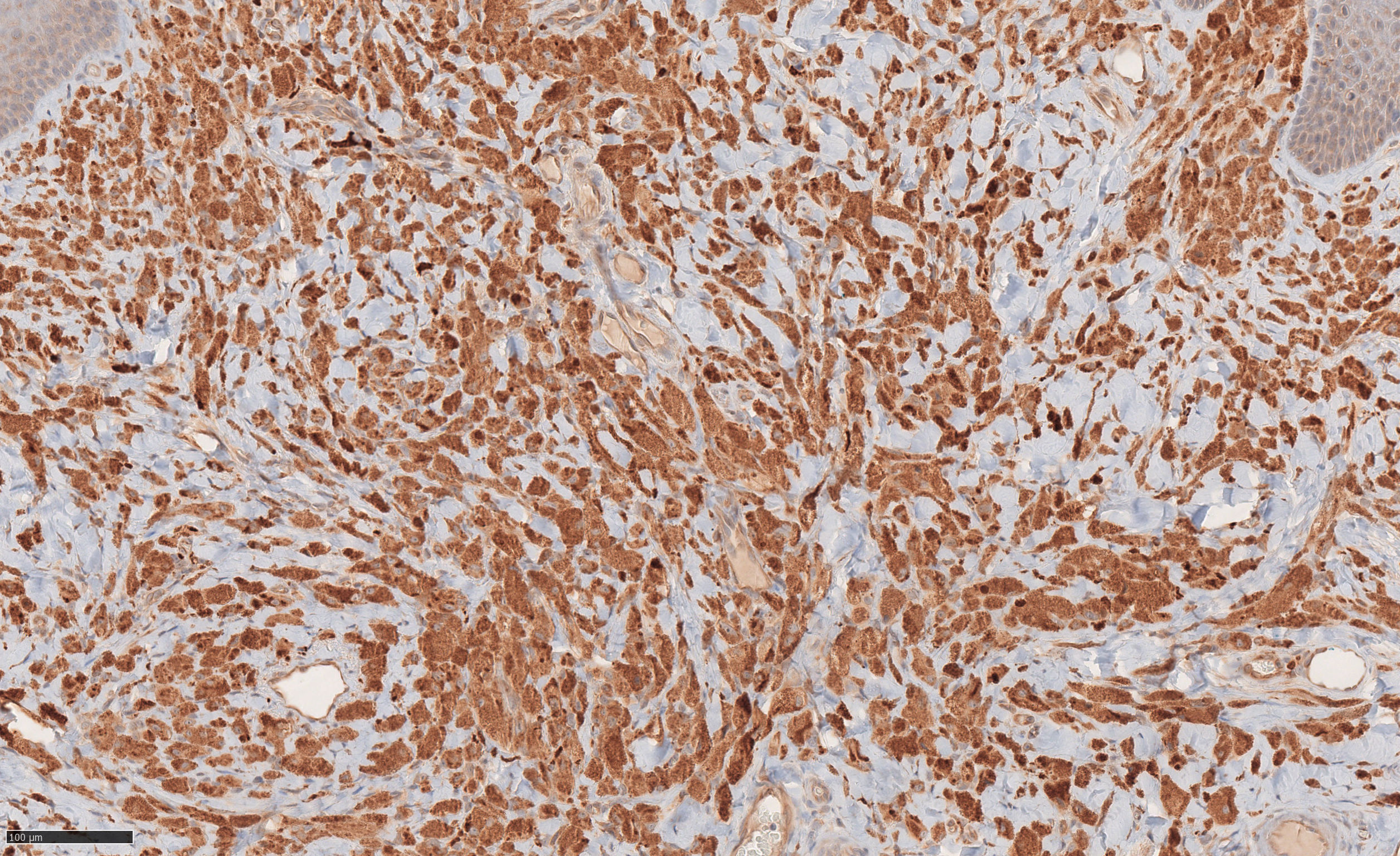

Supplement: Supplementary file 5 [file Image3.jpeg]
